# Supplementary material for: k-mer manifold approximation and projection for visualizing DNA sequences
Source: Genome Res. 2025 May;35(5):1234–46. doi: 10.1101/gr.279458.124 (PMC12047656; doi:10.1101/gr.279458.124)
Supplement: Supplement 8 [file Supplemental_Data_1.zip › Supplemental_Data_1/MLX_CATAT14N_S_3/MLX_CATAT14N_S_3_MDS.pdf]

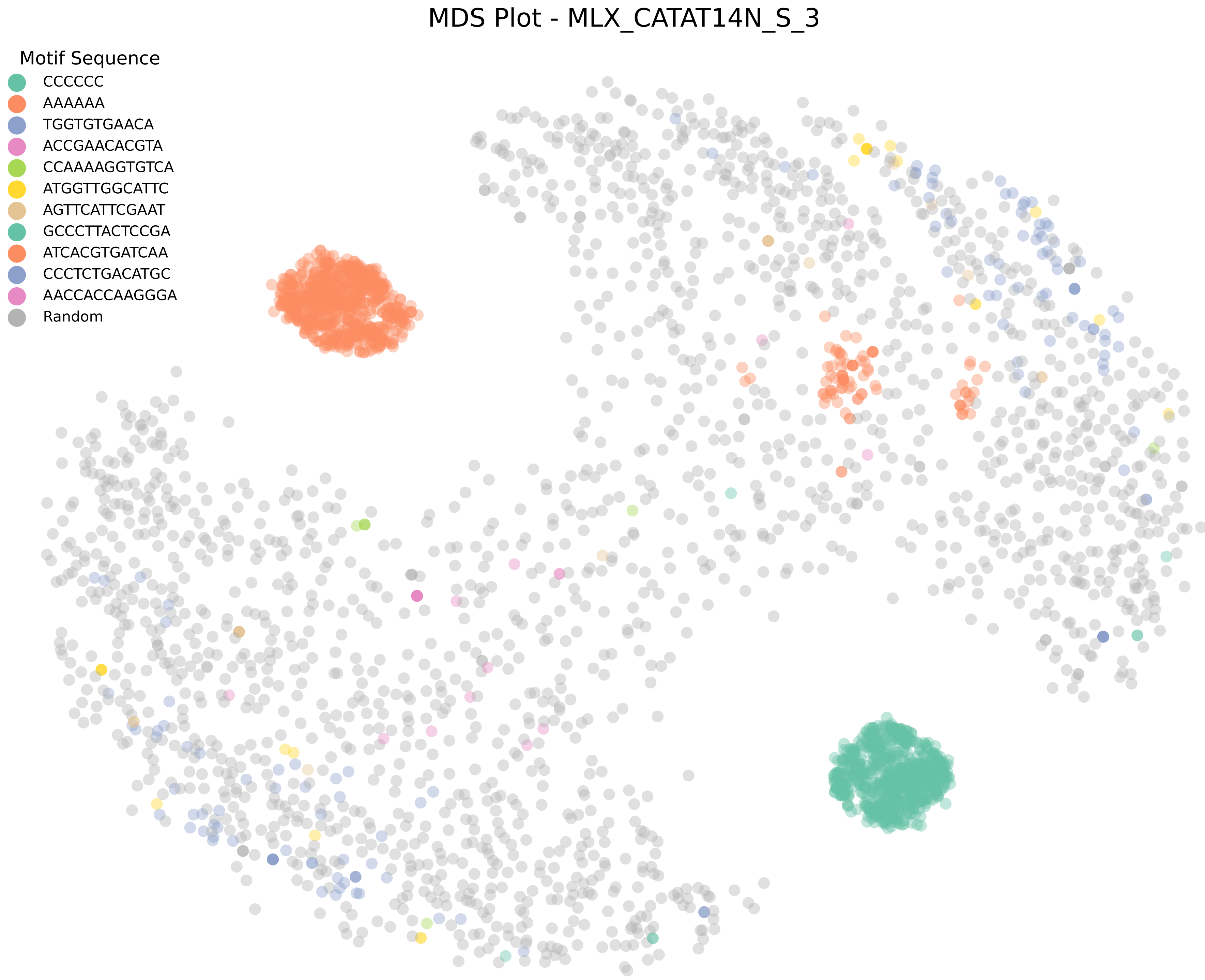
MDS Plot - MLX_CATAT14N_S_3
Motif Sequence
CCCCCC
AAAAAA
TGGTGTGAACA
ACCGAACACGTA
CCAAAAGGTGTCA
ATGGTTGGCATTC
AGTTCATTCGAAT
GCCCTTACTCCGA
ATCACGTGATCAA
CCCTCTGACATGC
AACCACCAAGGGA
Random
